# Spectral Analysis of ATP-Dependent Mechanical Vibrations in T Cells

**DOI:** 10.3389/fcell.2021.590655

**Published:** 2021-06-10

**Authors:** Ishay Wohl, Eilon Sherman

**Affiliations:** Racah Institute of Physics, The Hebrew University, Jerusalem, Israel

**Keywords:** power of diffusion, mechanical fluctuations, malignancy, DFT, microscopy

## Abstract

Mechanical vibrations affect multiple cell properties, including its diffusivity, entropy, internal content organization, and thus—function. Here, we used Differential Interference Contrast (DIC), confocal, and Total Internal Reflection Fluorescence (TIRF) microscopies to study mechanical vibrations in live (Jurkat) T cells. Vibrations were measured via the motion of intracellular particles and plasma membrane. These vibrations depend on adenosine triphosphate (ATP) consumption and on Myosin II activity. We then used spectral analysis of these vibrations to distinguish the effects of thermal agitation, ATP-dependent mechanical work and cytoskeletal visco-elasticity. Parameters of spectral analyses could be related to mean square displacement (MSD) analyses with specific advantages in characterizing intracellular mechanical work. We identified two spectral ranges where mechanical work dominated vibrations of intracellular components: 0–3 Hz for intracellular particles and the plasma-membrane, and 100–150 Hz for the plasma-membrane. The 0–3 Hz vibrations of the cell membrane that we measured in an experimental model of immune synapse (IS) are expected to affect the IS formation and function in effector cells. It may also facilitate immunological escape of extensively vibrating malignant cells.

## Introduction

Mechanical work inside living cells plays a significant role in cell physiology (Huang and Ingber, 2005; Tee et al., 2010). For instance, direct transport of intracellular constituents is conducted by molecular motors such as kinesin, dynein and myosin II (Manfred and Woehlke, 2003). Other indirect intracellular motor activities may control important biophysical parameters, including intracellular diffusivity (Brangwynne et al., 2009; Guo et al., 2014), entropy (Wohl and Sherman, 2019) and phase partitioning of cell content (Wohl and Sherman, 2019). Indirect motor activity may include the incoherent fraction of mechanical forces that are applied by molecular motors on multiple sites on the cytoskeleton (Guo et al., 2014). Thus, these incoherent forces impact the cytoskeleton. The cytoskeleton is an elastic mesh (Mizuno et al., 2007), and thus it transfers those forces to intracellular constituents, e.g., vesicles and organelles that are embedded within or adjacent to it. Applied forces on the cytoskeleton from tension generation by cortical actin may also influence the mechanical activity of the cytoskeleton (Chugh and Paluch, 2018).

Notably, mechanical work generates forces that are non-thermal and depend on ATP consumption. As a result of these forces, the cytoskeleton transfers mechanical work and augments the diffusion of intracellular particles (Brangwynne et al., 2009; Guo et al., 2014). It also increases intracellular entropy and decreases the partition of the intracellular content, as in liquid-liquid phase separation (LLPS) (Wohl and Sherman, 2019).

The plasma membrane (PM) is physically connected to the cortical actin (Salbreux et al., 2012). Thus, both thermal and active fluctuations of the actin mesh translate into corresponding fluctuations of the PM. In the case of T cells (studied here), their activation is an outstanding example of the significance of such PM fluctuations and their effect on cell biology and decision-making. T cells get activated upon specific triggering of their T-cell antigen receptor (TCR). Such triggering occurs when TCRs recognize their cognate ligands, namely antigens, presented on the major histocompatibility complex (pMHC) on the surface of antigen presenting cells (APCs) (Chakraborty and Weiss, 2014). Recently, it has been shown that the interactions between the TCR-pMHC and TCR activation depend on repeatedly applied perpendicular forces (Huppa et al., 2010; Ma and Finkel, 2010). Thus, the fluctuations described in this study could significantly contribute to the effective rates of TCR engagement and triggering, specificity of antigen recognition and cell activation.

Here, we aimed to study intracellular diffusion and intracellular mechanical work, as they occur in T cells. Specifically, the impact of non-equilibrium forces on intracellular particles motion enables the investigation of those forces by analyzing the dynamics of these particles. Intracellular diffusion motion is usually characterized as anomalous diffusion, for which the mean square displacement (MSD) is not linearly correlated to the time-lag of measurements (Granek and Pierrat, 1999; Caspi et al., 2000). The MSD equation is ⟨Δ*r*^2^⟩=*K*_α_*t*^α^, where *K*_α_ is the diffusion coefficient and α is the diffusion power. Finding these specific anomalous diffusion parameters does not usually facilitate the identification of the main cellular mechanisms that explains those results. The reason is that different underlying mechanisms may lead to similar anomalous diffusion *K*_α_ and α results (e.g., Golan and Sherman, 2017).

Three main mathematical models have been defined in relation to different cellular mechanisms that may govern the intracellular anomalous diffusion, including visco-elasticity, diffusion and percolation in a crowded environment and medium with traps or energetic disorder (Meroz et al., 2013). Fractional Brownian motion (FBM) is a model that is characterized by long-range temporal correlations and relates to diffusion motion in visco-elastic media. The model of Random walk on a fractal (RWF) enables to characterize diffusion motion or percolation in fractal media, such as crowded environment. Through the Continues Time Random Walk (CTRW) model, the particle diffusion is hindered by trapping events and binding interactions. The motion of the particle is characterized by a broad distribution of waiting times between jumps. In a wider definition, this model also applies to a medium with energetic disorder (Meroz et al., 2013).

The diffusion motion patterns of a particle that is stuck in a trap and will randomly gain enough energy to jump to another location may be similar to the diffusion motion patterns of a free particle that randomly gains a large amount of mechanical energy that will cause it to jump to a relatively remote location. Both situations are described by the CTRW model. Accordingly, the impact of mechanical energy on intracellular diffusion is likely to cause the anomalous diffusion patterns to better match that model. A distinctive difference between the CTRW model and the other FBM or RWF models is that the CTRW model describes a non-ergodic process, while FBM and RWF describe ergodic processes (Jeon et al., 2011; Meroz et al., 2013). Accordingly, if a break in ergodicity could be demonstrated while analyzing diffusion motion in living cells, it is reasonable to assume that in that situation the CTRW model and its related underlying mechanisms are dominant and better explain the cellular condition (Meroz et al., 2013).

Following that, an analytic framework of intracellular diffusion motion that combines the effects of spatial fluctuations with ergodicity breaking should clearly capture the impact of intracellular mechanical work on the anomalous diffusion. Such an analysis should be able to discern underlying mechanisms of intracellular diffusion motion that may yield similar anomalous diffusion parameters (*K*_α_ and α) but differ in their ergodicity.

The power spectral density (PSD) analysis of a wide range of time-dependent parameters was studied in many fields of science, including physics, biophysics, geology, weather science, etc. (Krapf et al., 2018). Often, the PSD has the form: μ_s_(f,∞)=A/f^β^ (Krapf et al., 2018). This prevalent PSD function has been defined analytically for diverse situations including chaotic Hamiltonian systems (Geisel et al., 1987), periodically driven bi-stable systems (Shneidman et al., 1994), fluctuations of a phase-separating interface (Delfino and Squarcini, 2016), Brownian Diffusion (BD) (Krapf et al., 2018), and multiple models of anomalous diffusion (Krapf et al., 2018, 2019; Sposini et al., 2019). Except from theoretical studies, PSD of diffusion motion was investigated mainly in simulations and basic experimental setups incorporating quantum dots (Houel et al., 2015) or trajectories of tracers in artificial crowded fluids (Weiss, 2013), but not in live cells. When investigating vibrations in live cells, a combined contribution to the PSD of two components has to be considered: First, a homogenous and random (white-noise-like) contribution due to thermal forces. Second, a periodic or incoherent contribution due to inhomogeneous mechanical work. The second component is naturally related to a break in ergodicity and could be more readily distinguished while analyzing intracellular modes of vibration utilizing PSD calculations.

Spectral analysis of the dynamics live cells constituents may have the advantage of providing better insight into the biophysical mechanisms behind their anomalous diffusion and ergodicity breaking, especially in regard to intracellular mechanical work.

Here, we utilized a relatively simple spectral analysis framework for the exploration of intracellular diffusion and intracellular mechanical work. This framework is based on Discrete Fourier Transform (DFT) of temporal position changes of intracellular constituents. This framework then serves to analyze the intracellular diffusion of intracellular particles (e.g., vesicles or other small organelles) and fluctuations of cell diameter in live Jurkat cells, before and after ATP depletion. From the PSD results of the motion of cell constituents, we define parameters that reflect intracellular mechanical work. We show that cells under normal (physiological) conditions are active and produce significant extent of mechanical work. This work is diminished in the same cells that become non-active after ATP depletion. Next, we explore intracellular mechanical work over a wide spectrum of time-scales and frequencies. We identified two spectral ranges where mechanical work dominated vibrations of intracellular components: 0–3 Hz for intracellular particles and the plasma-membrane, and 100–150 Hz for the plasma-membrane. Such vibrations of the cell membrane are expected to affect the formation and function of the immune synapse by effector cells. Thus, we studied the membrane vibrations of Jurkat cells in an experimental model of the immune synapse using total internal reflection fluorescence (TIRF) microscopy. Indeed, we identified ATP-dependent membrane fluctuations at the model synapse, esp. below 3 Hz. These mechanical fluctuations of the cell membrane may also affect T cell recognition of extensively vibrating malignant cells. We expect that spectral analysis of intracellular vibrations and motion will become a useful tool for characterizing cell condition and activity in health and disease.

## Results

### Spectral Analysis of Temporal Fluctuations of Large Intracellular Particles Is Related to Intracellular Diffusivity and Intracellular Mechanical Work

The cytoskeleton is an elastic polymeric mesh that spans the intracellular volume with a mesh size of around 50 nm (Guo et al., 2014). The elastic cytoskeleton mesh is surrounded by a crowded viscous intracellular gel-like medium. These two constituents largely make the two-component visco-elastic cellular content (Brangwynne et al., 2009). Notably, the mechanical response of the intracellular medium is mainly elastic and less viscous (Guo et al., 2014) with low Reynolds number (Cartwright et al., 2009). The energy due to vibrations in this elastic cytoskeleton has the value of: *E*_*mechanical*_ = 0.5*kA*^2^ + *E*_*loss*_, where *E*_*loss*_ is the (relatively small) dissipated energy, *k* is the equivalent spring constant of the system, and *A* is the amplitude of the motion. Monitoring movements of an intracellular particle (like a vesicle) that is significantly larger than the cytoskeleton mesh size (50 nm) can reveal the movements of the adjacent cytoskeleton mesh. Spectral analysis of this particle movement (i.e., its change in position over time) will express multiple modes of vibrations of the adjacent cytoskeleton mesh. Each vibration mode of this mesh has a mechanical energy level of approximately 0.5*kA*^2^. The integral of the spectrum of vibrations represents the approximated total mechanical energy of the measured part of the cytoskeleton mesh in the specified spectral range. Monitoring movements of multiple intracellular particles and averaging the spectral analysis results of these movements enable insight into the mechanical energy and work of the entire cytoskeleton and cellular system.

Thermal agitation forces and incoherent intracellular mechanical forces (which are a by-product of directed forces that are utilized for cell physiology), both act on the cytoskeleton. Together, they contribute to the cytoskeletal modes of vibrations. These vibration modes can then be revealed by monitoring embedded particles inside the mesh for their diffusion motion. Spectral analysis of the diffusion motion of these particles can be related to the modes of vibration and mechanical energy of the adjacent cytoskeleton.

**FIGURE 1:**
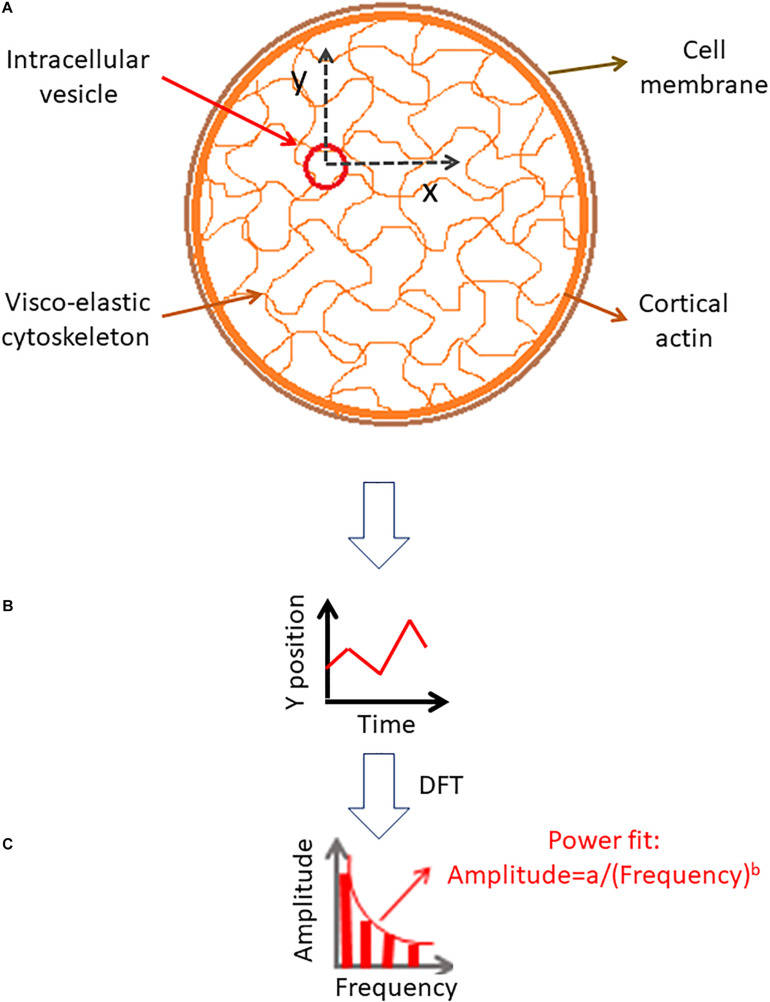
Analysis of intracellular particle motion embedded insidethe cytoskeletal mesh. **(A)** Particle motion in x and y dimensions. **(B)** Demonstration of the particle position change in time in one dimension. **(C)** Discrete Fourier Transform (DFT) of the particle position serial results [as shown in panel **(B)**].

In order to explore these relations we consider the diffusion motion of an intracellular particle embedded in the elastic cytoskeleton mesh as illustrated in Figure 1. The change in particle position over time could be analyzed by DFT to produce the particle’s amplitudes of spatial fluctuations for the corresponding spatial dimensions (x or y). These amplitudes of spatial fluctuations represent the different modes of vibrations that determine the combined mechanical energy of the particle and adjacent elastic cytoskeletal mesh.

To further investigate these spectral amplitudes of spatial fluctuations, we consider the three following aspects:

1. The summation (integral) of all powers (the squared amplitudes), which relates to the total mechanical energy of the particle-elastic mesh system in the specified spectral range. Each power represents a specific amplitude of vibration (*A or Amplitude*) and its corresponding mode of mechanical energy (namely, *E*_*mechanical*_ = 0.5*kA*^2^).
2. The vibration spectrum is fitted with a power equation for each set of spectral results for each cell and condition:
  (1) $$Amplitude_{estimated}=a/{\left(frequency\right)}^{b}$$
  The relation of *a* and *b* parameters of the power fit to the diffusion and mechanical work characteristic are further studied below.
3. The extent to which the actual spectrum of amplitudes fits to the model equation of estimated power. In other words, we quantify the magnitude of the sum of squared errors (SSE) that relates the spectrum to its power fit equation. Then, the correlations of SSE values to the intracellular diffusion and mechanical work characteristic are investigated.

**FIGURE 2:**
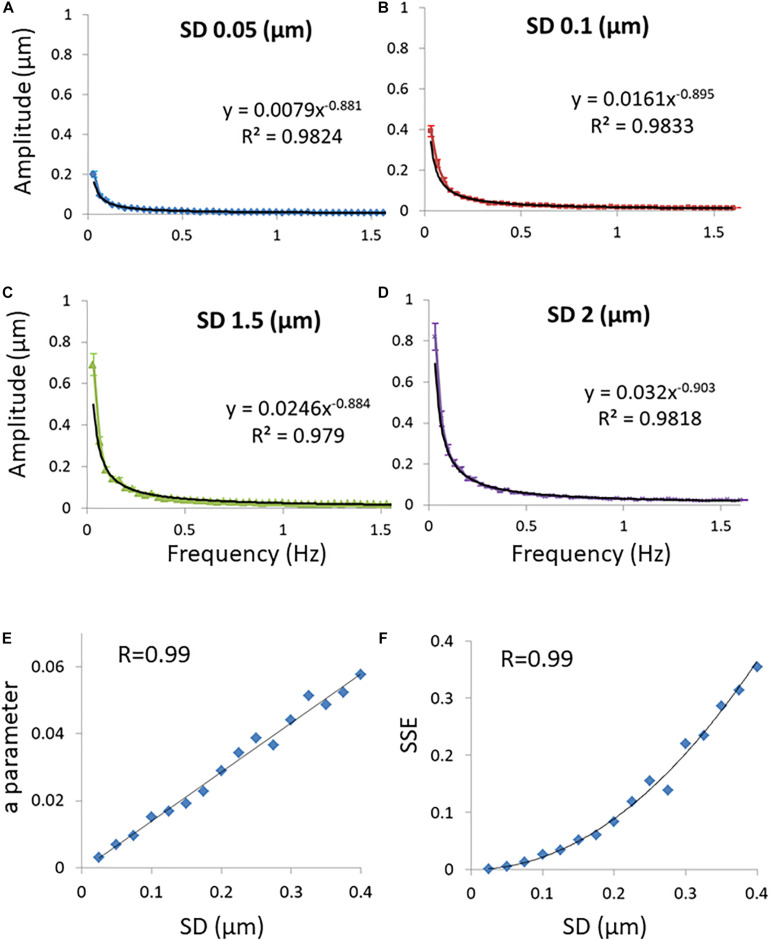
Simulations results. **(A)** Discrete Fourier Transform (DFT) of a simulated particle position change in time when the probability density function’s (PDF) of translocations are Gaussian and the SD of translocations is 0.05 μm. Power fit parameters of the DFT results are added. **(B)** The same as **(A)** except that the SD of translocations is 0.1 μm. **(C)** The same as **(A)** except that the SD of translocations is 0.15 μm. **(D)** The same as **(A)** except that the SD of translocations is 0.2 μm. 50 simulations were performed for each condition described in panels **(A–D)**. **(E)** The correlation between the SD of translocations and the *a* parameter of the power fit results (*y* = *ax^b^*) in each condition of SD. **(F)** The correlation between the SD’s of translocations and the sum of squared errors (SSE) of the power fit in each condition of SD. Error bars in panels **(A–D)** are SEM.

A general diffusion process can be characterized by its typical probability density function (PDF) of translocations for each corresponding time-lag, and following that, by the statistics of MSDs. First we analyze the condition of Brownian diffusion, for which α=1. The PDF of translocation for each time-lag in this case is Gaussian. We simulated the movement of particles undergoing normal diffusion with different diffusion coefficients (represented by the different standard deviations of the PDF of translocation Gaussians; Figures 2A–D). In this simulation, calculating the spectra of amplitudes of fluctuations (of position changes in time) reveals that the compatibility of a power fit model to these spectral results is high. It also shows that the *a* parameter of the power fit [*Amplitude*_*estimated*_ = *a*/(*frequency*)^*b*^] is correlated to the standard deviation (SD) of PDF of translocations (Figure 2E) and accordingly, to diffusivity. Following this simulation of normal diffusion we conclude that the SSE of the power fit is also correlated to the SD of PDF of translocations and diffusivity (Figure 2F).

The characteristic PDF of a diffusing particle (along with its parameters *K*_α_ and α) can be related to the spectral analysis results of its position changes in time. For an ergodic diffusion process, the PDF of translocations in a time-lag that corresponds to the interval between measurements reveals the statistic of the sequential translocation steps of that diffusive object. Knowing this statistic of translocations does not allow us to reconstruct the exact trajectory of that particle during measurements. Still, one can anticipate the different amplitudes of spatial fluctuations and their correspondent statistical frequencies from the given PDF of translocations. The knowledge of the different amplitudes of spatial fluctuation with their corresponding frequencies of occurrence is generally equivalent to the results of the DFT analysis of the position fluctuations of that same particle over time. In this analysis, the different frequency-dependent amplitudes of the DFT represent the different amplitudes of the particle’s spatial fluctuations (i.e., translocations). Similarly, the corresponding frequencies of these amplitudes (in the DFT) represent the equivalent probability of occurrence of these translocations (in the PDF). Thus, it is expected that the DFT results of this particle diffusion movements will be related to its PDF of translocations and accordingly, to its diffusion parameters.

Following that, the possible analytical relations between the DFT power fit parameters *a* and *b* and the PDF parameters *K*_α_ and α in conditions that included anomalous or non-ergodic diffusion were explored and described in Supplementary Material. Power fit parameter *a* was found to be correlated to the PDF parameter *K*_α_ (this compatible with the simulation experiment results presented in Figure 2E) while *b* parameter of the power fit was found to be correlated to the PDF α parameter in anomalous ergodic diffusion process. In a non-ergodic diffusion processes with a brake in symmetry that correlation between *b* parameter and α is expected to break also. Parameter *b* as being more sensitive to the brake in symmetry of the diffusion process will increase more in comparison to α.

In living cells: the applied forces that relate to intracellular mechanical work may be directional in contrary to the perfect random and symmetric thermal forces or the elastic forces of the relatively symmetric cytoskeleton mesh. Those forces due to intracellular mechanical work will break the ergodicity and the symmetry of the affected diffusion motion in cells. Following that, the high *b* values in this condition of non-equilibrium non-ergodic process may be the result of two contributions to *b*. The first contribution to *b* relates to large translocations (α) and the shape of the PDF. This adds to a second contribution to large translocations that relates to the breaking of ergodicity and symmetry due to mechanical work.

To summarize the expected influence on the *a* and *b* parameters of the power fit by adding mechanical work to the cellular system:

• The *a* parameter increases due to the increase in *K*_α_ and diffusivity associated with active cells.
• Values of *b* also increase due to the increase in α (power of diffusion), while added mechanical work breaks ergodicity and further increases *b* values.

According to these arguments, the product value of *a*⋅*b* could be used to differentiate active cells from non-active ones.

In an ergodic diffusion process, SSE values of the power fit formula that fit the spectral amplitudes of position temporal fluctuations are correlated to diffusivity, as demonstrated in Figure 2F. This result may be explained by the assumption that in a more dynamic system the errors from expected values should be more significant. As the dynamics of the system or variance of translocations is higher, the variance of variance (or the forth moment) will be higher as well. It is reasonable to assume that in an active, non-ergodic system the applied forces are not perfectly random and symmetric as thermal forces. Accordingly, the amplitudes spectrum in this case will be less “smooth.” Thus, the SSE values of the power fit will increase further relative to the basic levels of SSE values due to the degree of dynamics of the particles and errors of measurements.

### Spectral Analysis of Intracellular Particles Position Fluctuations in Jurkat T Lymphocytes Before and After ATP Depletion

In this section, we present the results of the spectral analysis of position fluctuations in time of intracellular particles in Jurkat T lymphocytes, each cell before and after ATP depletion. Specifically, we explore the relations of these results to diffusion parameters (*K*_α_ and *α*) that were calculated for the same cells and conditions. Depletion of cellular ATP was induced by 30 min incubation with 0.2 μM of the mitochondrial complex 1 inhibitor Rotenone (Sigma-Aldrich,St. Louis, MO, United States) together with 10 mM glycolysis inhibitor 2-deoxy-D-glucose (Sigma-Aldrich,St. Louis, MO, United States) (Guo et al., 2014). For the visualization and monitoring of intracellular objects, including vesicles, we employed Differential Interference Contrast (DIC) microscopy (Supplementary Figure 1a). Cells were imaged repeatedly every 0.3 s, with a total of 100 images acquired for each cell. For intracellular particle tracking we utilized the ImageJ plugin MultiTracker (Kuhn lab, the University of Texas at Austin) (Wohl and Sherman, 2019; Wohl et al., 2019). First, the x and y positions of each identified particle at each point of time (determined by the MultiTracker plug in) were recorded (Supplementary Figures 1b–e). Then, the DFT of these time-dependent position changes were calculated for each spatial dimension. The average DFT amplitudes of all moving particles in each cell under each condition were determined for the x and y spatial dimensions. A fit to a model of power series (*y* = *a*/*x^b^*) was conducted for the DFT amplitudes results such that the parameters *a*, *b*, and *SSE* of the power fit could be obtained. See section “Materials and Methods” for further details on imaging and analyses.

From the position results of the detected intracellular particles, we also calculated the MSD values for time-lags form 0.3 to 3 s (with a 0.3 s gradual increase). The average MSD values were determined for that series of time-lags for each cell before and after ATP depletion. These values were fitted to a model of power series to determine the corresponding *K*_α_ and *α* values for the underlying diffusion process in these cells.

As expected, the *K*_α_ and *α* values were higher in the normal active cells, as compared to the same cells after ATP depletion (*K*_α_: 0.023 μm^2^/s vs. 0.013 μm^2^/s with *p* < 10^–4^. *α*: 0.924 vs. 0.809, with *p* < 10^–3^).

To estimate ergodicity in the cells, we employed a basic principle which implies that in an ergodic system the distribution of translocations of a specific trajectory is not dependent on its spatial location. Following that, the distributions of translocations of all trajectories in a perfectly ergodic system are similar. In our experiment, the distribution of translocations of each trajectory or a particle is reflected by this particle’s MSD results. Evaluating the heterogeneity of all particles MSD results in a cell (SD of MSD results in that cell) will produce an estimation of the ergodic level in that cell system (Krapf et al., 2018). When we compared in that way the level of ergodicity in the active cells to the level in the same cells after ATP depletion, we found (as expected) that the level of ergodicity in active normal cells is lower than in non-active ATP-depleted cells (SD of MSD’s = 0.026 μm^2^ in normal cells vs. SD of MSD’s = 0.018 μm^2^ in ATP-depleted cells, *p* = 0.01).

**FIGURE 3:**
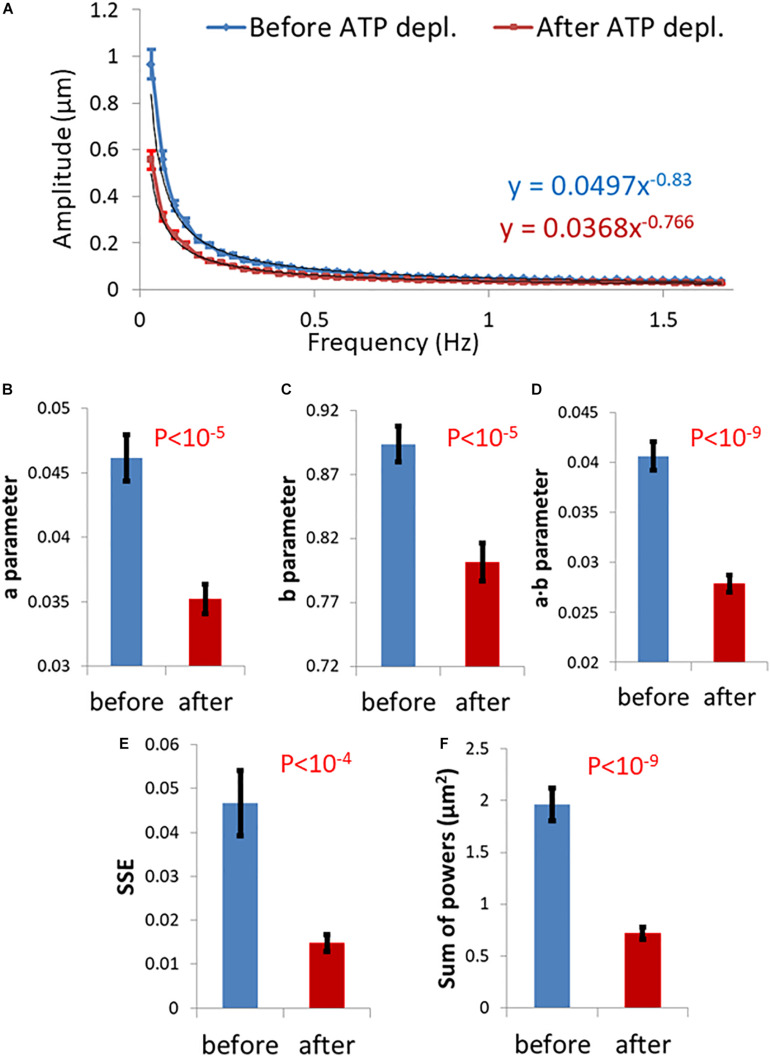
Differential Interference Contrast (DIC) microscopy results of intracellular particles motion in live cells before and after adenosine triphosphate (ATP) depletion analyzed by amplitude spectral density (ASD) and power spectral density (PSD). **(A)** ASD analysis results in (*n* = 29) cells before (blue line) and after (red line) ATP depletion and the corresponding power fit parameters. **(B–E)** Power fit parameters: *a*, *b*, *a⋅b*, and *SSE* for the ASD analysis results in the cells [in **(A)**] before and after ATP depletion. **(F)** Power fit parameter “*Sum of powers*” for the PSD analysis results in the cells [in **(A)**] before and after ATP depletion. Error bars in panels **(A–F)** are SEM.

We summarize in Figure 3 the results of the DFT analysis of particles position changes in living Jurkat cells before ATP depletion and DFT results of the same living cells after ATP depletion (that inhibits cellular active mechanical work). As expected: *a*, *b*, *SSE*, and the *sum of all powers* are all higher in active live cells as compared to the same cells after ATP depletion.

**FIGURE 4:**
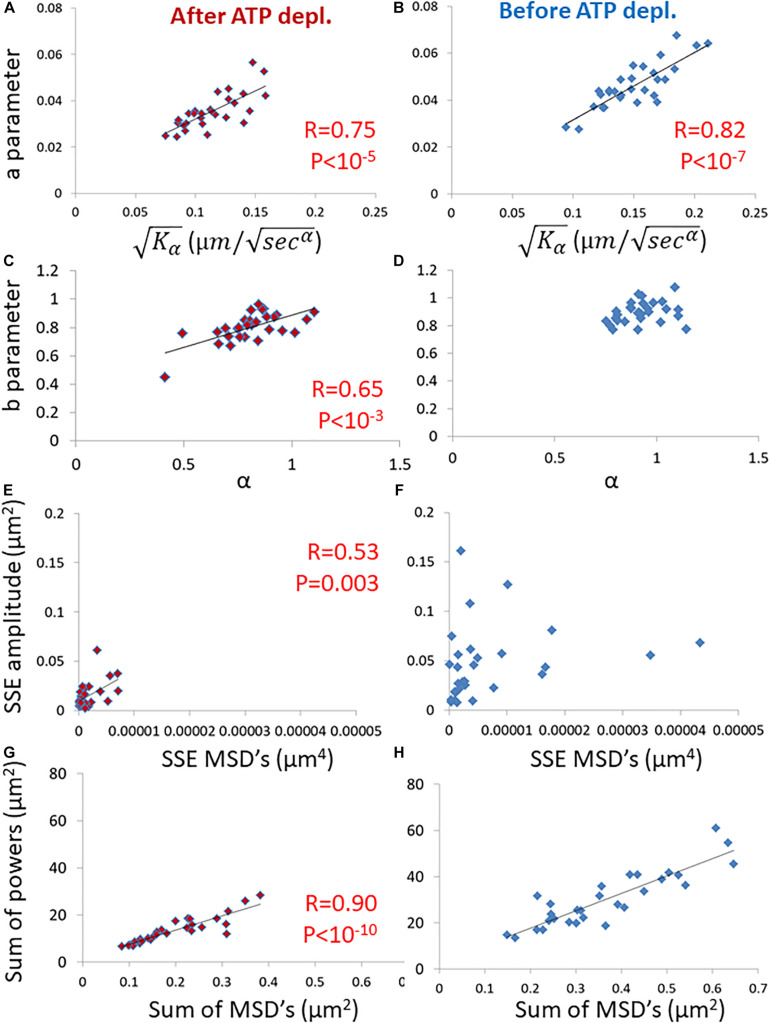
Correlation of amplitude spectral density (ASD)/ power spectral density (PSD) parameters and mean square displacement (MSD) parameters in live cells before and after adenosine triphosphate (ATP) depletion. **(A)** The correlation between the *a* parameter of ASD analysis and $\sqrt{K_{\alpha }}$ of MSD analysis in ATP-depleted cells. Analyses relate to DIC microscopy results of intracellular particles motion of shown in Figure 3 (*n* = 29). **(B)** The correlation between the *a* parameter of ASD analysis and $\sqrt{K_{\alpha }}$ of MSD analysis in cells before ATP depletion. **(C)** The correlation between the *b* parameter of ASD analysis and α of MSD analysis in ATP depleted cells. **(D)** The correlation between the *b* parameter of ASD analysis and α of MSD analysis in cells before ATP depletion. **(E)** The correlation between the SSE parameter of ASD analysis and SSE of power fit to MSD analysis in ATP depleted cells. **(F)** The correlation between the SSE parameter of ASD analysis and the sum of squared errors (SSE) of power fit to MSD analysis in cells before ATP depletion. **(G)** The correlation between “Sum of powers” parameter of PSD analysis and sum of MSD’s parameter (from MSD analysis) in ATP depleted cells. **(H)** The correlation between “Sum of powers” parameter of PSD analysis and sum of MSD’s parameter (from MSD analysis) in cells before ATP depletion. *R*- and *P*-values of significant correlations are presented in the corresponding correlations images.

Next, we evaluated the ability of these parameters, derived from spectral analysis of position fluctuations, to capture different aspects of particle motion, esp. in comparison to the prevalent anomalous diffusion parameters *K*_α_ and *α*. Specifically, we studied the correlation between these two groups of parameters, as shown in Figure 4.

Strikingly, the two groups of parameters were significantly correlated in ATP-depleted cells (Figures 4A,C,E,G). In non-active cells, most of mechanical forces are thermal and random. Thus, we expect that: the *a* parameter will be related to *K*_α_, the *b* parameter will be related to *α* and *SSE* of the fit to the amplitudes will be related to the *SSE* of fit to the MSD values (as discussed in the previous section).

On the other hand, for cells before ATP depletion, there was no correlation between *b* and *α* probably due to the break in ergodicity (as also discussed above). These cells were physiologically intact and active, and produced mechanical work. Therefore, the *b* parameter values may express the break in ergodicity that is typical for living active cells.

Moreover, in active cells the correlation between *SSE* of the fit of amplitudes and the *SEE* of the fit of MSD’s also seemed to break. This is probably due to the addition of forces that relates to intracellular mechanical work. These forces may act in a similar time-scale and frequencies to our observations and may directly influence the spectrum of position fluctuations related to these frequencies.

The *sum of powers* of the spectral analysis was found to be correlated to the *sum of all MSD* values under both cell conditions (Figures 4G,H). The sum of powers of the particles’ spatial fluctuations over time may represent a summation of all modes of vibrations (in this time-scale) of these particles that are embedded in an elastic medium. Therefore, this summation of all powers relates to the total mechanical energy of this system, namely the mechanical work plus thermal energy when the system is under non-equilibrium. It relates only to thermal energy when the system is in equilibrium. The sum of MSD values represents the diffusivity of these particles. The mechanical energy and diffusivity are expected to be correlated in cellular systems both under equilibrium and non-equilibrium conditions, as shown in a previous work (Wohl and Sherman, 2019).

**FIGURE 5:**
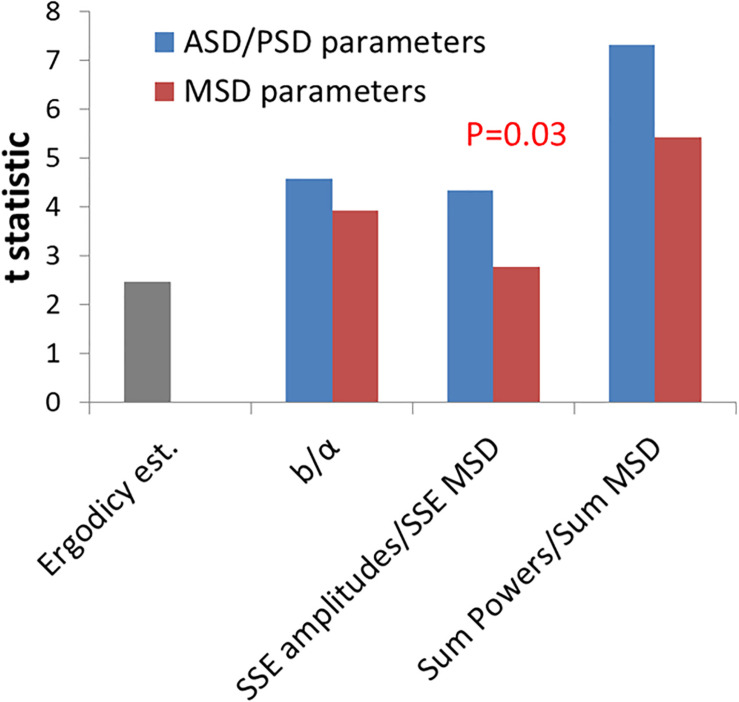
Comparing the discrimination power of power spectral density (PSD)/amplitude spectral density (ASD) vs. mean square displacement (MSD) analyses for intracellular mechanical work. T-statistics values for the difference between cells before ATP depletion and the same cells after ATP depletion according to the two groups of parameters: ASD/PSD analysis based parameters in compare to MSD analysis based parameters. Corresponding parameters from the two groups are compared: the *b* parameter vs. α, sum of squared errors (SSE) of amplitudes vs. SSE of MSD’s, and “Sum of powers” vs. Sum of MSD’s. The t-statistics value for the ergodic test (described in “Results” section) is also presented. The *P*-values for the difference in t-statistics values between ASD/PSD parameters and the corresponding MSD parameters was calculated according to *t*-test for paired two samples and was found to be 0.03.

Next, we further wanted to test if these new parameters, which relate to the amplitudes or powers of temporal position fluctuations, have a better discriminative ability to differentiate between active working cells and non-active ATP-depleted cells in comparison to the classical diffusion parameters of *K_α_ and α*. We analyzed the t-statistic results that have been obtained while using each parameter to differentiate the two physiological conditions (Figure 5). The t-statistic values of each parameter when comparing normal active cells to the same cells after ATP depletion reflect the discriminative strength of that parameter to create two groups of results that are statistically diverse. The t-statistic values of the new parameters are higher than the t-statistic values of classical diffusion parameters. This difference is statistically significant as summarized in Figure 5. The discriminative ability or t-statistic of the parameter for ergodic estimation alone is the lowest (Figure 5; left bar).

We conclude that the new DFT-derived parameters may detect better the increase in mechanical energy that characterizes active and physiologically normal cells (in contrast to non-active ATP-depleted ones), in comparison to the classical diffusion parameters of *K_α_ and α*.

### Spectral Analysis of Temporal Fluctuations in Cell Diameter Before and After ATP Depletion

**FIGURE 6:**
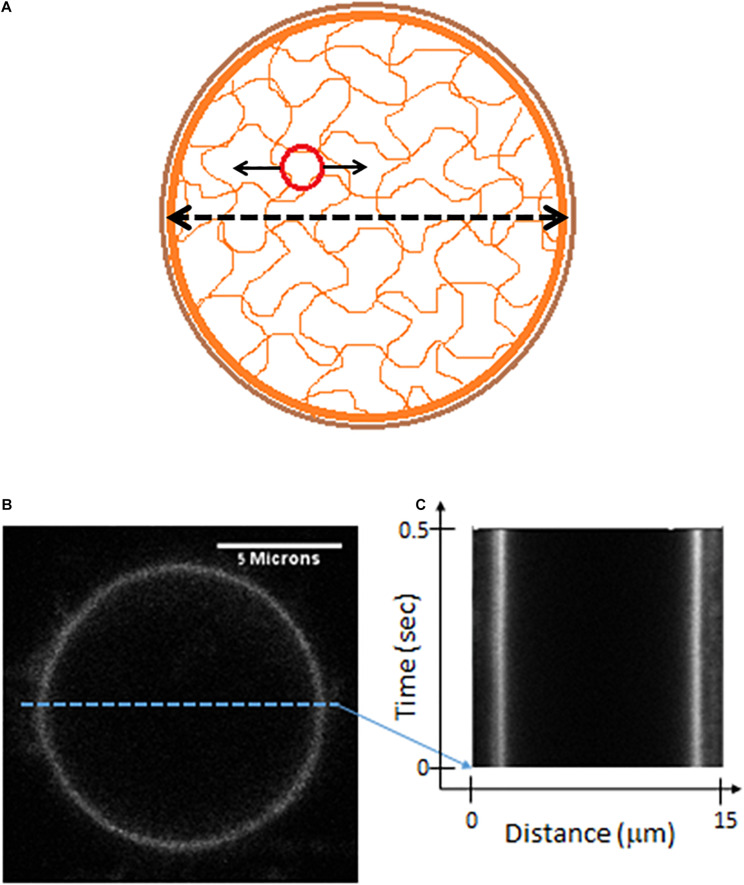
Live cells Measurements of cell diameter fluctuations. **(A)** A scheme of the cell showing mechanical relations of its constituents. The possible mechanical relation between intracellular cytoskeleton fluctuations (that may be detected by the motion of imbedded particles) and the fluctuations of cytoskeleton borders or the cortical actin (that may be detected by following changes in cell diameter). **(B)** Confocal imaging of a live Jurkat cells, stained with CD45. **(C)** A kymograph showing fast (2 ms) and repetitive line-scans across the cell membrane at its apparent mid-section, as shown in panel **(B)**.

In active cells the mechanical work of the elastic cytoskeleton augments the motion of intracellular particles (Mizuno et al., 2007; Brangwynne et al., 2009; Guo et al., 2014; Wohl and Sherman, 2019). By that it increases the amplitude of the particles’ modes of vibrations and mechanical energy. This mechanical work of the elastic cytoskeleton mesh on particles that are embedded within must have a co-effect on the dynamics of the elastic mesh borders, which are intimately related to the cell membrane. In other words, the actively vibrating and elastic cytoskeletal mesh that augments the motion of embedded particles will also produce matching vibrations of its boundaries, which are mechanically coupled to the cell membrane by the cortical actin (schematically illustrated in Figure 6A; Salbreux et al., 2012; Chugh and Paluch, 2018). Following that, we hypothesized that monitoring fluctuations in cell diameter should exhibit similar spectral patterns to the spatial fluctuations of intracellular particles.

**FIGURE 7:**
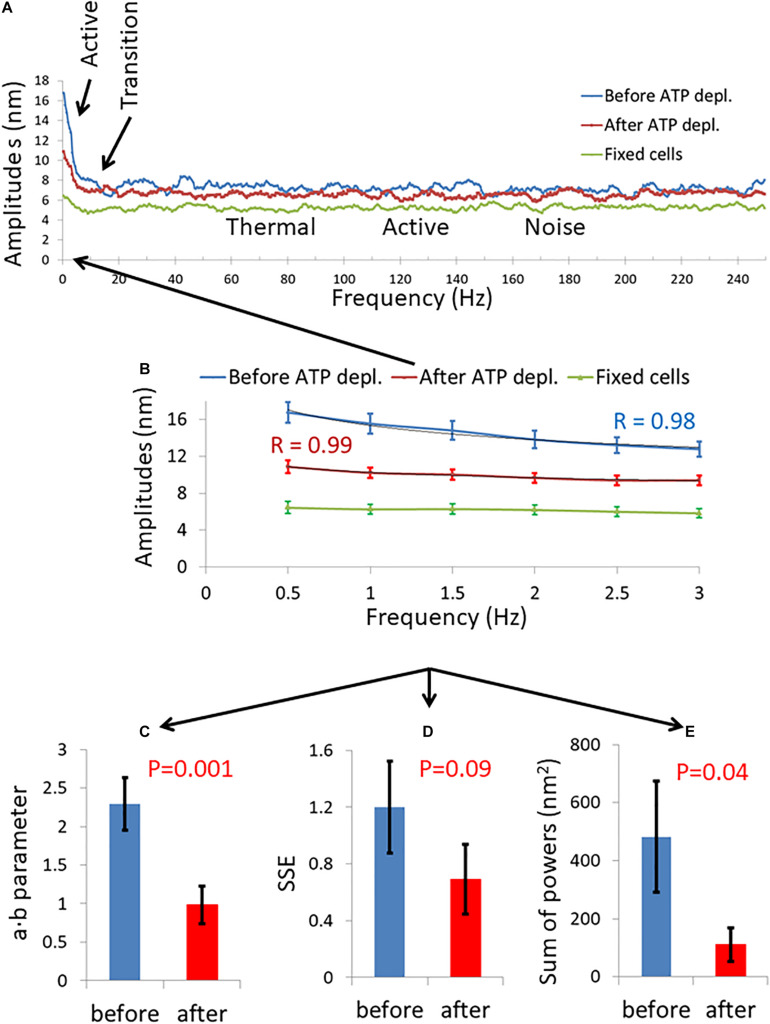
Amplitude spectral density (ASD) and power spectral density (PSD) analysis results of live cells diameter fluctuations before and after adenosine triphosphate (ATP) depletion. **(A)** Average ASD analysis results in cells before (blue line) and after (red line) ATP depletion. Control average ASD results of 20 fixed cells are also presented (green line). Specific ranges of frequencies according to their dominant mechanism of fluctuations are highlighted. *N* = 14 cells. **(B)** Focusing on ASD results in the frequencies range of 0–3 Hz out of the total frequencies range presented in image **(A)**. R values for the power fit to the ASD results are also presented. **(C)** Average power fit parameter *a⋅b* results of the ASD analysis presented in image **(B)** before and after ATP depletion. **(D)** Average power fit parameter sum of squared errors (SSE) results of the ASD analysis presented in image **(B)** before and after ATP depletion. **(E)** Average power fit parameter “Sum of powers” results of the PSD analysis before and after ATP depletion. Error bars in **(B–E)** are SEM.

In order to test this hypothesis we first highlighted the cell membrane via fluorescent staining of CD45, an abundant surface glycoprotein in T cells (Donovan and Koretzky, 1993). Then we conducted fast and repetitive line-scans across the cell membrane at its apparent mid-section by confocal scanning microscopy (Figure 6B). Acquisition time for a single line-scan was 2 ms, using a pixel width of 50 nm. The repetitive line-scans could then be presented as a kymograph (Figure 6C). Cell membrane position was determined according to the pixel with the highest intensity. Cell diameter fluctuations were analyzed in each Jurkat cell before and 30 minute after ATP depletion. After DFT analysis, average amplitudes of each frequency were calculated for each experimental condition. The data were then smoothed using a moving average window of 10 data points in the spectra (i.e., 5 Hz). The average amplitudes of 14 Jurkat cells, each cell before and after ATP depletion, are presented in Figure 7. As a control, the average amplitudes of 20 Jurkat cells after fixation are presented as well. As can be seen in Figure 7A there are two distinct frequencies ranges for power fit: <3 Hz and >3 Hz. The first range of frequencies (<3 Hz) is compatible with the range of frequencies that have been explored in the previous section, utilizing repeated DIC images of intracellular particles. Concentrating on amplitudes in the frequency range <3 Hz (Figure 7B) reveals that these amplitude-spectra are similar to the amplitude-spectra obtained for DIC images of intracellular particles. According to Figure 7E, the *sum of powers* parameter is reduced after ATP depletion and the power fit parameters, namely *SSE* and *a*⋅*b*, are also reduced (Figures 7C,D). As could be assumed, it seems that the elastic cytoskeleton modes of vibrations that augment the motion of large intracellular particles (meaning, larger than the mesh size) also impact the motion of the borders of that elastic mesh that are adjacent to the cell membrane. In that way, modes of vibration of the cell diameter relate to modes of vibration of intracellular particles. Both of these vibration types reflect intracellular mechanical work that is done on the elastic cytoskeleton.

### Inspecting the Higher Frequencies in the Power Spectra of the Cell Diameter Fluctuations Before and After ATP Depletion

**FIGURE 8:**
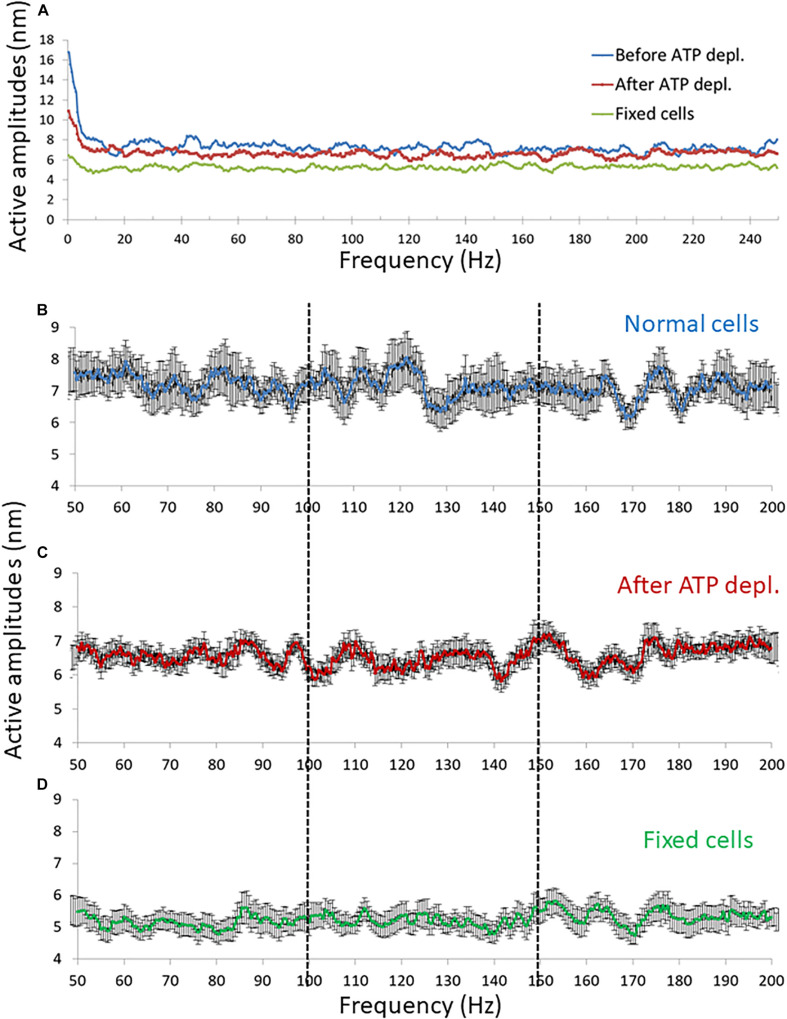
Amplitude spectral density (ASD) results in high frequencies (>50 Hz). **(A)** Average ASD results in normal non-treated cells (*N* = 14), adenosine triphosphate (ATP) depleted cells (*N* = 14) and dead fixated cells (*N* = 20). **(B–D)** Average ASD results in the frequencies range between 50 and 200 Hz in normal non-treated cells **(B)**, ATP depleted cells **(C)**, and fixated dead cells **(D)**. Error bars in panels **(B–D)** are SEM.

Our fast confocal imaging of the cell diameter and its fluctuations enabled us to examine also the modes of vibration of this diameter at relatively high frequencies. Specifically, we studied the amplitudes of fluctuations of cell diameter at 50–200 Hz in cells before and after ATP depletion and also in fixed cells (Figure 8). From these results, it seems that in the frequency range between 100 and 150 Hz, the amplitudes are less random relative to the amplitudes in the other frequency ranges.

In an ideally viscous medium in equilibrium, the spectrum of thermal forces on a particle is equivalent to white noise and independent of frequency. Thus, the power spectrum of spatial fluctuations of such a particle should be completely random and non-correlated. If correlations in the particle motion appear due to extra thermal forces, as elastic forces in the medium or forces due to mechanical work, then the amplitude spectrum of that particle motion is expected to be less random. Autocorrelation analysis of the amplitude spectra will lead to higher values and a decrease in decay with increased frequency lags. Therefore, autocorrelation analysis may differentiate an amplitude-spectrum that is more typical to ideal Brownian process or to noise from an amplitude-spectrum that is more typical to elastic forces or mechanical work.

**FIGURE 9:**
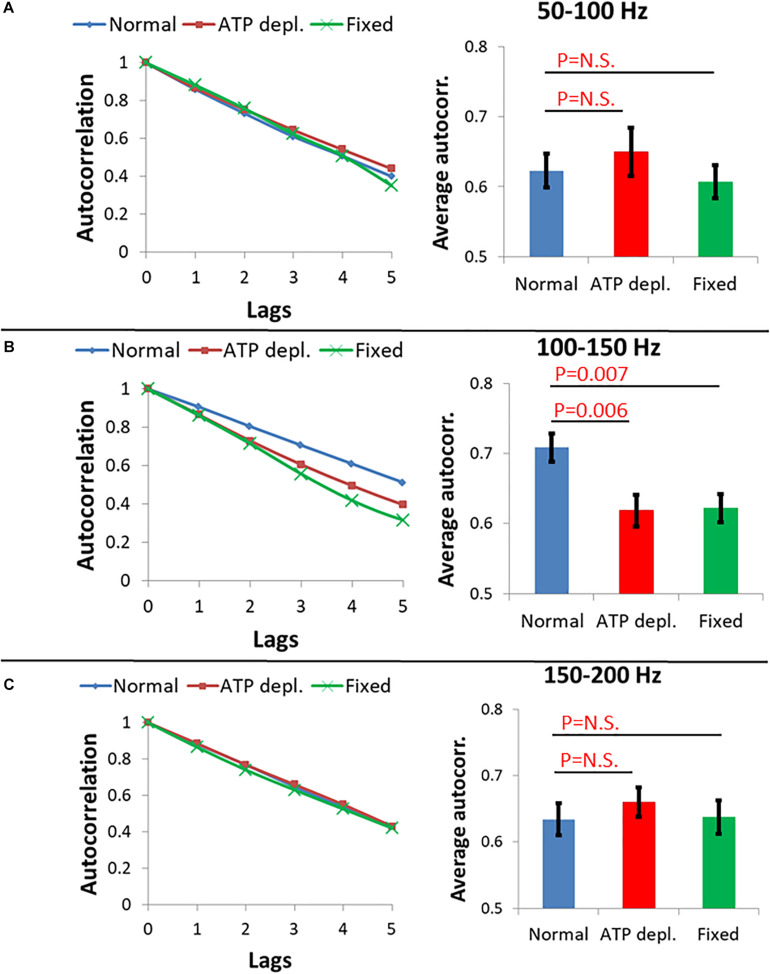
Average autocorrelation results of Amplitude spectral density (ASD) analysis. **(A)** Average autocorrelation results of ASD in normal non-treated cells (*N* = 14), adenosine triphosphate (ATP) depleted cells (*N* = 14) and dead fixated cells (*N* = 20) in the frequencies range between 50 and 100 Hz. Results relate to the spectral data in Figure 8. On the right the average autocorrelation values of 1–5 lags in each cellular condition with their SD of mean are presented. **(B)** Average autocorrelation results of ASD in the normal non-treated cells, ATP depleted cells and dead fixated cells in the frequencies range between 100 and 150 Hz. On the right the average autocorrelation values of 1–5 lags in each cellular condition with their SD of mean are presented. **(C)** Average autocorrelation results of ASD in the normal non-treated cells, ATP depleted cells and dead fixated cells in the frequencies range between 150 and 200 Hz. On the right the average autocorrelation values of 1–5 lags in each cellular condition with their SD of mean are presented.

Following this concept, three ranges of frequencies of the amplitude-spectra of the cells were analyzed for autocorrelation: 50–100 Hz, 100–150 Hz, and 150–200 Hz. In each range of frequencies, the average autocorrelation results for each lag, for each cell and for each cellular condition are presented in Figure 9. According to Figure 9A, at the 50–100 Hz range, the decay of the autocorrelation function was similar and pronounced for the fixed cells, normal and ATP-depleted cells. At the next frequencies range of 100–150 Hz, the fixed and ATP-depleted cells have similar and more pronounced decay relative to the autocorrelation function decay of normal cells. At the last frequencies range of 150–200 Hz, the decay was similar and pronounced under all cells conditions.

We assume that in ATP-depleted cells no significant mechanical work is produced. Following that assumption, it seems that the difference in the decay between normal and ATP-depleted cells in 100–150 Hz may be due to a larger extent of mechanical work in normal active cells that reduces the randomness of their amplitudes of vibrations. If elasticity was the main contributing factor in this frequency range, the decay of autocorrelation in fixed cells and ATP-depleted cells are not expected to be similar. This is since the mechanical characteristics of the intracellular medium are very different under these two conditions. Last, at the frequency ranges of 50–100 Hz and 150–200 Hz, autocorrelation decay functions under all conditions are pronounced and similar. This indicates that the measured powers in this frequency range may represent thermal agitation or noise of the measurement system.

Differences in the shape of the amplitude-spectra (Figure 8, compare panels b with c and d) and related autocorrelation analyses (Figure 9B) suggest that intracellular mechanical work can be observed and related primarily to the 100–150 Hz frequency range.

### Fluctuations of Jurkat Cell Membrane in a Model of an Immune Synapse

**FIGURE 10:**
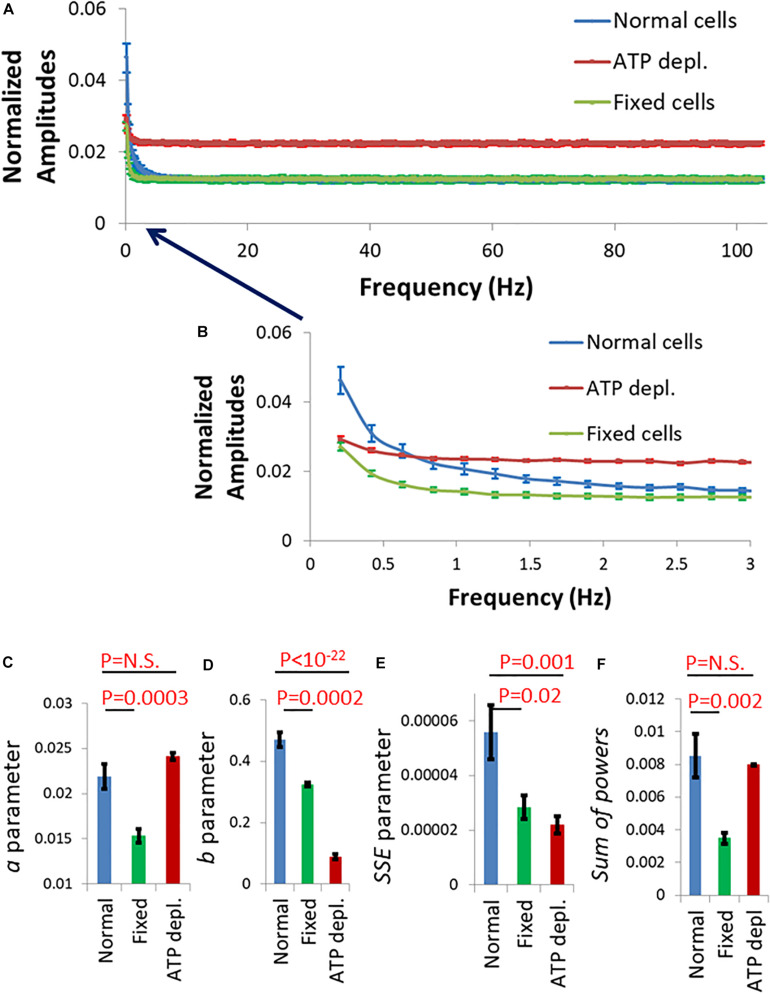
Fluctuation of Jurkat cell membrane in a model of the immune synapse imaged by TIRF microscopy. **(A)** Discrete Fourier Transform (DFT) results of fluctuations of normalized intensity of anti-CD45 antibodies labeled with Alexa647, which highlighted the plasma membrane of Jurkat cells. The cells adhered to coverslips coated with anti-CD3ε. Amplitudes of normal cells (*N* = 30) are in blue, adenosine triphosphate (ATP) depleted cells (*N* = 33) are in red and fixed cells (*N* = 24) are in green. **(B)** Frequency range of 0–3 Hz of the amplitudes in **(A)**. Power fit parameters: *a* **(C)**, *b* **(D)**, sum of squared errors (*SSE*) **(E)**, and *Sum of powers* **(F)** results of the amplitudes presented in **(B)**. Error bars in panels **(A–F)** are SEM.

So far, we have described intracellular fluctuations as captured by the motion of intracellular particles or by the cell boundaries. The fluctuations of the cell membrane may have an impact on the formation and function of the immune synapse that forms between T cells and antigen presenting cells (APCs) (Huppa et al., 2010; Ma and Finkel, 2010). To study such a possible impact, we measured fluctuations of Jurakt cells at their interface with coverslips coated with αCD3ε antibodies. Such coverslips often serve as a model for the immune synapse, as the cells spread on the coverslips and get robustly activated (Balagopalan et al., 2011). The membrane of the cells was stained with an αCD45 primary antibody, labeled with Alexa647 and imaged by time-lapse TIRF microscopy. Imaging in TIRF enabled sub-diffraction sensitivity of the intensity signal to membrane fluctuations along the Z-axis (perpendicular to the interface) (Balagopalan et al., 2011). For each cell, 1,000 images were captured in a time lag of 4.8 ms between each sequential image. Live cells were measured without and after ATP depletion (as described in the section “Materials and Methods”), while fixed cells served for control. In each cell, we chose for analysis a squared region of interest (ROI) of 121 pixels at the cell interface with the coverslip (see section “Materials and Methods”). The temporal fluctuations of the normalized fluorescence intensities were analyzed by DFT for each pixel in that ROI. The amplitudes were then averaged for each frequency for all the pixels of an ROI. The averaged DFT results of each ROI (or cell) could then be compared between cells and conditions and analyzed by the power fit parameters as presented in Figure 10.

The amplitude spectral density (ASD) results for frequencies >3 Hz (and up to 100 Hz) were similar for normal cells and cells after fixation (Figure 10A; green and blue curves). Measured fluctuations in fixed cells were likely due to thermal motion and noise of the measurement system. The thermal fluctuations depend on the mechanical properties (as elasticity or stiffness) of the measured system. In our synapse model, the stiffness of the coverslip governed the mechanical properties of the system and the related thermal motion in fixed and live normal cells. Otherwise, and as expected, we could not detect active fluctuations at that frequency range in live cells (see Figure 7A). The relatively higher level of thermal fluctuations in ATP-depleted cells is probably due to an interruption of the immune synapse and some disconnection of cells from the stiff αCD3-coated coverslip.

At frequencies <3 Hz, membrane fluctuations were significantly higher in normal active cells than in fixed cells and in ATP-depleted cells (Figure 10B; compare blue curve to green and red curves). Also, the power fit parameters were significantly higher in normal cells relative to fixed cells (Figures 10C–F). Due to the suggested partial disconnection of the ATP depleted cells from the coverslip their thermal/baseline fluctuations were higher and that increased their *a* and *sum of powers* parameters values (Figures 10C,F). Parameters *b* and *SSE* are less sensitive to the extent of adhesion of the cells to the coverslip since they depend mostly on the shape of the power-fit curve. Thus, *b* and *SSE* capture better the conditions of low intracellular mechanical work in those ATP depleted cells. These results suggest that ATP-dependent cytoskeletal motion significantly contribute to membrane fluctuations at low (<3 Hz) frequencies; and that these fluctuations directly modify the interface of the cells with a TCR-activating surface that mimics the immune synapse.

## Discussion

Here we studied active modes of vibrations of the cytoskeleton and plasma membrane in lymphocytes. Specifically, we analyzed spatial fluctuations of intracellular particles and of the cell diameter utilizing DFT analysis. The cytoskeletal motion was studied by monitoring the motion of intracellular large particles, larger than the cytoskeleton mesh size of around 50 nm (Guo et al., 2014). Such particles are typically embedded inside the cytoskeleton (Luby-Phelps, 2000). Their motion reflects cytoskeletal fluctuations due to the mechanical coupling between these particles and the surrounding elastic mesh. Cytoskeletal fluctuations could then be also reflected in fluctuations of the cell membrane and its diameter. The amplitudes of vibration of the cytoskeleton were related theoretically and experimentally to the extent of intracellular mechanical work. We could further compare mechanical vibrations in physiologically intact cells in comparison to ATP-depleted cells or cells after treatment with blebbistatin. Focusing on relative changes in the results under these various conditions largely cancels out possible contribution of extracellular effects, which are unchanged between these measurements.

The extent of mechanical vibrations in lymphocytes, especially the mechanical vibrations of the plasma membrane, could significantly impact the immune synapse (Huppa et al., 2010; Ma and Finkel, 2010). Thus, we demonstrated the existence of active, ATP-dependent vibrations of the cell membrane of Jurkat T cells after spreading and activation in an experimental model of an immune synapse. In that way, the extent of cellular mechanical vibrations may influence the ability of T lymphocytes to create stable immune synapse and to generate adequate immune response.

There are two main approaches to characterize the complex intracellular medium. If viewed as a highly complex solution, then the motion of intracellular particles could be naturally analyzed in terms of time-dependent translocations and diffusivity. Still the intracellular content can also be viewed as a two component elastic gel; i.e., an elastic polymeric mesh made of the actin cytoskeleton, immersed in crowded viscous gel. In this case, the medium would be more intuitively analyzed in terms of the spectrum of its vibrations.

The random motion of particles has been theoretically and experimentally investigated through their modes of vibration using PSD analysis. The PSD analysis is classically calculated by first performing a Fourier transform of each individual trajectory x(t) [or y(t)] over the finite observation time T and then averaging the spectral results for a statistical ensemble of all possible trajectories (Krapf et al., 2019). In some theoretical types of anomalous diffusion this analysis is not integrable. In such cases, the PSD analysis was adopted to use the Fourier transform of the autocorrelation function of the random process, since autocorrelation is integrable. This enabled to characterize the spectral contents of that non-integrable process according to Wiener–Khinchin theorem (Sposini et al., 2019). In our experiments, the number of measurements was relatively large (100), yet finite. Thus, the particles’ displacement results were analyzed by DFT, while the conversion to the autocorrelation function seemed unnecessary. In each cell the results of many particles or trajectories were averaged (average number of trajectories for a cell was 39 with SD of 16) which enabled us to rely on a statistical ensemble for the amplitude spectral density (ASD) or PSD calculations, as in the classic way for calculation of these spectra.

In the case of Brownian motion the relation between powers of fluctuations and the related frequencies could be described by a power-law equation in the form:

(2) $$\mu_{s}=\frac{cK_{\alpha }}{f^{2}}$$

where μ_*s*_ stands for the power, *f* is the related frequency, *K*_α_ stands for the diffusion coefficient and *c* is a constant (Krapf et al., 2019). From that equation the amplitude of fluctuations, *A_s* can be defined as

(3) $$A_{s}=\frac{\sqrt{cK_{\alpha }}}{f^{1}}$$

In the case of FBM sub-diffusion, when α < 1, the power of the frequency (*f*) is changed to α+1 (compare to a value of 2 in Brownian motion) (Krapf et al., 2019; Sposini et al., 2019).

Our experimental results in living cells follow these theoretical equations. Indeed, we find that the PSD (or ASD) results can be accurately fitted with a power-law equation (Eq. 1). The parameter *a* derived from the fits to the experimental amplitudes were linearly correlated to the square root of diffusivity and K_α_ results (Figures 4A,B). This is expected from the theoretical relation $a=\sqrt{cK_{\alpha }}$. The values of the power *b* of the fit to the ASD results were relatively close to 1, as suggested by Eq. 3 for FBM. Nevertheless, the differences of *b* from 1 may be attributed to the major differences between intracellular motion and Brownian motion due to elasticity, fractal media, mechanical work and break in ergodicity (Luby-Phelps, 2000; Meroz et al., 2013; Guo et al., 2014).

Aside from the fit parameters of *a* and *b*, we considered two additional parameters: *SSE* of the power fit and *sum of powers* of the PSD analysis. We found that all of these ASD- and PSD-related parameters were sensitive to intracellular mechanical work. This work augmented the cytoskeletal vibrations via a non-ergodic process.

We found that the ASD- and PSD-related parameters could better distinguish mechanically active cells from non-active ATP-depleted cells, as compared to the regular MSD-based anomalous diffusion parameters (Figure 5). The lower sensitivity of the MSD-based anomalous diffusion parameter α to respond to intracellular mechanical work may be related to fact that ATP-depletion reduces the elasticity of the cytoplasm of living cells. Accordingly, the value of α in ATP-depleted cells may be influenced by two opposing effects: First, an increase in α value due to the decrease in elasticity upon ATP depletion (Guo et al., 2014); Second, a decrease in α due to the decrease in motivated random forces (Brangwynne et al., 2009). On the other hand, the corresponding parameter *b* of the ASD analysis is influenced also by the break in ergodicity that accompanies the increase in intracellular mechanical work. This sensitivity of *b* to non-ergodic processes may improve its ability to detect the effect of the increase in intracellular dynamics, associated with intracellular mechanical work.

Incoherent forces due to intracellular mechanical work may be applied at different locations on the cytoskeleton, each with its own frequency. Such forces are expected to make the ASD results of cytoskeletal fluctuations more complex, lowering the quality of a fit to a relatively simple power-law model. In this situation the system could be characterized as having relatively high energetic disorder, which directly relates to lower ergodicity. In contrast, ASD results of the same network experiencing only thermal forces (i.e., “white noise” forces that don’t have any frequency preference) will more accurately follow a fit of a power-law model. Accordingly, *SSE* values are higher in normal active cells in comparison to non-active ATP-depleted cells. Again, these differences are due to the addition of mechanical forces and break in ergodicity in active cells. Measuring the parameter of *Sum-of-powers* is a relatively direct way to evaluate the mechanical energy of the vibrating system—in this case the cytoskeleton.

The calculation procedure of the ASD or PSD fluctuation parameters seems to be simpler and more automatic in comparison to the calculation of MSD parameters. MSD analyses require taking statistical measurements of multiple displacement results that relate to different time-lags for each particle.

The cytoskeletal vibrations could be monitored by motion of particles that are embedded within it but also by the motion of it borders- namely, the cortical actin and the adjacent cell membrane. Analyzing fluctuations in cell diameter by ASD and PSD calculations that were conducted in the same cells before and after ATP-depletion revealed compatible results with the ASD and PSD analysis of intracellular particles motion. The power fit parameters of cell diameter fluctuations *a*⋅*b* and the *sum of powers* were higher in normal active cells, as compared to the same cells after ATP depletion.

Our confocal microscope line-scan imaging of fluctuations of the cell diameter enabled to conduct ASD analyses over a wide range of frequencies. Thus, we could define several specific ranges of frequencies, each with a dominant underlying mechanism (Figure 7A). These mechanisms control the amplitude of the cytoskeletal fluctuations in their related spectral range, as follows: At 0–3 Hz cytoskeletal fluctuations are governed by incoherent fraction of ATP-dependent molecular motors (such as myosin II) forces, as suggested by Guo et al. (2014). This mechanism may also explain our DIC results of the motion of intracellular particles that were measured in similar lower frequencies (Figure 3). To further support this suggested mechanism we conducted similar experiments to those presented in Figure 3 with cells treated with the myosin II inhibitor blebbistatin (Supplementary Figure 2). The effects on intracellular motion of blebbistatin treatment and ATP depletion were similar (compare Figure 3 and Supplementary Figure 2). These findings support the role of myosin II motors in the generation of our described intracellular mechanical work and active motion. At the successive range of frequencies below 100 Hz, thermal agitation dominates. This was also suggested by Guo et al. (2014), and is also supported by our current findings (Figure 9A). Interestingly, analysis of cell diameter fluctuations at 100–150 Hz indicated that active, ATP-dependent mechanical fluctuations likely dominate in this frequency range. We propose that active mechanical tension fluctuations and tension generation of the cortical actin may explain these results (Wohl et al., 2020), but this assumption requires further validation.

We note that the T cell surface is covered with mobile microvilli. Microvilli mobility has been shown to depend on actin remodeling, and occurs over time scales of seconds to tens-of-seconds (Cai et al., 2017). Since our measurements of cell diameter relies on the identification of a stain of the plasma membrane, they could in principle capture some of the microvilli dynamics and interfere with our diameter measurements. However, our spectra are focused on relatively faster processes (of 0.5–3 Hz) than actin remodeling (Walker et al., 2020). Moreover, we show in previous studies that our measured spectra correlate with forces and volume changes that occur at the borders of the cell with similar spectra to our current measurements (Wohl et al., 2020). These findings support that active cell fluctuations dominate our measured spectra. Still, a possible contribution from microvilli dynamics on our measurements cannot be completely ruled out at the lowest frequencies.

TCR activation has been shown to be a dynamic process, in which the TCR-pMHC bond is repeatedly ruptured and reconnected by perpendicular forces to the immune synapse plane. Rupture forces acting on the TCR-CD3-pMHC bond promote conformational changes of the TCR chains that promote TCR activation (Ma and Finkel, 2010). Specifically, these conformational changes enables exposure and phosphorylation of immunoreceptor tyrosine-based activation motifs (ITAMs) on the TCR intracellular chains and further propagation of T cell activation signals (Hwang et al., 2020). Here, we have shown that cytoskeleton vibrations may result in corresponding vibrations of the cell diameter and plasma membrane. The matching of the TCR-CD3-pMHC bond strength and pulling forces at the synapse could enable specificity of the TCR response. Repeated triggering of the TCR by cell vibrations could further provide sensitivity and specificity to this response (Ma and Finkel, 2010). The described force strength and frequency through this process are around 10 pN and 1 Hz (Limozin et al., 2019). According to our results of cell diameter fluctuations (Figure 7B), the perpendicular component of the cell membrane fluctuations at 1 Hz has an amplitude of ∼16 nm. From previous AFM measurements (Wohl et al., 2020), the tension fluctuations of the (Jurkat) cell surface at 1 Hz have an amplitude of ∼0.001 μN/μm. Multiplying the tension fluctuations of 0.001 μN/μm and spatial movements of 16 nm produces forces of ∼16 pN due to this motion. Thus, we conclude that the scales of forces and frequencies related to our measured membrane fluctuations are suitable for activating engaged TCRs at the immune synapse. In that way, lymphocyte vibrations may control or interfere with the T cells’ ability to respond to important immunological stimuli.

**FIGURE 11:**
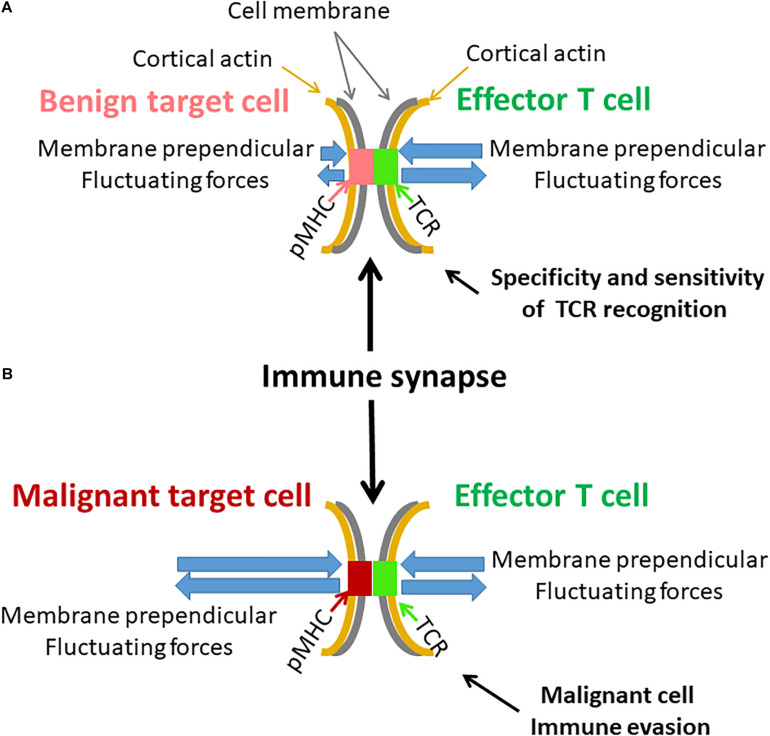
Possible effects of cell membrane fluctuations on T cell function. **(A)** An illustration of an immune synapse between an effector T cell with its specific TCR and a benign target cell with a complimentary pMHC. Both cell membranes and their attached cortical actin are mechanically vibrating but to a different extent. Resultant perpendicular forces act on the TCR-pMHC complex, leading to its modified lifetime. The benign target cell membrane is relatively firm and stable. The more pronounced membrane fluctuations of the effector T cell could effectively produce repeated and precise rupture forces on the TCR-pMHC complex to generate effective cellular response. **(B)** A similar illustration of an immune synapse between effector T cell and a malignant target cell. The malignant target cell membrane and its underlying cortical actin are less firm and fluctuate more relative to benign cells [as in panel **(A)**]. The fluctuations of the effector T cell are milder relative to the membrane fluctuations of the malignant target cell. Thus, the TCR-pMHC complex is subject to incoherent forces, abrogating precise and organized activation of the TCR and the effector T cell. This may further lead to possible immune evasion of the malignant cell.

Here, we studied intracellular and membrane vibrations in Jurkat cells that originated from human leukemic T cells. Malignant cells typically have increased active mechanical fluctuations at relatively low frequencies (below 3 Hz) (Lau et al., 2003; Guo et al., 2014). They also have a softer surface (Xu et al., 2012; Hayashi and Iwata, 2015), which depends mainly on the degree of cortical actin tension. If indeed fluctuations of the T cell membrane contribute to sensitivity and specificity of TCR-pMHC interactions, these properties of (non-adherent) malignant cells could hinder the ability of the T cell to properly recognize and react against the malignant cells. Specifically, the T cell may not be able to accurately evaluate the affinity of the TCR-pMHC bond, and to create repeated and sufficient TCR deformations for maximal triggering of the TCR. By this mechanism, transformed cells may escape immune surveillance and killing by cytotoxic T cells (this mechanism is illustrated in Figure 11).

We conclude that spectral analysis (either ASD or PSD) may provide a simple and effective technique to study active cellular vibrations and the overall mechanical activity of cells. Active vibrations of the cell membrane may influence lymphocyte ability to respond to immunological cues and may further enable malignant cells to escape immunological surveillance.

## Materials and Methods

### Materials

Complete Medium (medium): RPMI-1640, DMEM medium, heat-inactivated fetal calf serum (FCS), penicillin, streptomycin, glutamine, sodium pyruvate, and HEPES obtained from Biological Industries (Kibbutz Beit Haemek, Israel). Rotenone and 2-deoxy-d-glucose from Sigma-Aldrich (St. Louis, MO, United States). CD45 proteins were purchased from BioLegend. Anti-human CD3 from eBioscience Inc. (Thermo Fisher Scientific). Blebbistatin was purchased from Sigma-Aldrich (St. Louis, MO, United States).

### Cell Line

Jurkat (human leukemic) E6.1 (CD4^+^) T cells were a kind gift from the Samelson lab at the NIH. Jurkat cells were maintained in RPMI-1640 medium supplemented with 10% FCS, 100 U/ml penicillin, 100 μg/ml streptomycin, 2% glutamine, 2% sodium pyruvate and 2% HEPES. Cells were maintained in completely humidified air with 5% CO2 at 37°C.

### Immunostaining

CD45 proteins were labeled using mouse anti-human primary antibodies conjugated to Alexa647 fluorophore (BioLegend, 304056). Labeling procedure followed the manufacturers’ protocols. Briefly, 0.5 μg of mouse anti human anti-CD45 monoclonal antibody conjugated to Alexa647 was added to 500 × 10^3^ cells suspended in FACS buffer for 45 min on ice. Cells were then washed in phosphate buffered saline (PBS) for three times and suspended in imaging buffer (RPMI without phenol red, 10% FBS, 25 mM HEPES).

### Sample Preparation

Coverslip preparation was as follows: coverslips (#1.5 glass chambers, iBidi) were washed with acidic ethanol at room temperature (RT) for 10 min and dried at 37°C for 1 h. Coverslips were than incubated at RT for 15 min with 0.01% poly-L-lysine (Sigma) diluted in water. This was followed by washing and drying of the coverslips at 37°C for 1 h. For the immune synapse model experiment the poly-L-lysine covered coverslips were incubated for 2 h at 37°C with 10 μg/ml anti CD3 antibodies diluted in PBS. Than the chambers were washed three times with PBS and left with PBS till the application of cells. Finally, cells were suspended in imaging buffer at a concentration of 1 million and 100,000–500,000 cells and were applied onto coverslips.

### Cells Fixation

Paraformaldehyde (PFA) 4% was added to the cells medium while on the coverslips in a ratio of 3/2 for 45 min incubation afterword all liquid were gently aspirated and replaced with imaging buffer (RPMI without phenol red, 10% FBS, 25 mM HEPES).

### Treatment of Jurkat Cells With Blebbistatin or Rotenone and 2-Deoxy-D-Glucose

Upon completion of measurements in all the cells and after recording the location of each cell, blebbistatin 10 μM or Rotenone 0.2 μM and 10 mM 2-deoxy-d-glucose were added to the cells medium. The samples were than incubated for 30 min on the microscope stage. At the end of incubation, each cell was measured again according to its recorded location. We excluded from analysis moving cells that may have changed their location during measurements.

### Microscope

Differential interference contrast (DIC) image stacks were taken with FV-1200 confocal microscope (Olympus, Japan) equipped with an environmental incubator (temperature and CO2) using a 60×/1.42 oil objective.

Confocal microscopy: Jurkat cells were imaged using an Abberior Expertline confocal/STED microscope (Abberior Instruments, Göttingen, Germany), mounted on a TiE Nikon microscope and operated by the Imspector software (v0.13.11885; Abberior Instruments, Göttingen, Germany). The cells were excited using a 638 mn pulsed laser (90 ps) 2 mW/cm2 at 50% power for x-t live cell experiments. Samples were imaged with a (CFI-SR-HP) Apochromat TIRF X100 NA 1.49 oil immersion objective (Nikon Instruments). Image stacks were generated by taking 1,000 serial images with acquisition time of 2 ms for frames of unidirectional 300 pixels (50 nm pixel size, 5 μs pixel dwell time). The reflection light was detected using an APD with a band-pass filter of 650–720 nm and a pinhole setting of 1.1 Airy units. Each line was scanned once.

TIRF microscopy: Jurkat cells were imaged using a TiE Nikon microscope. The cells were excited using a 647 mn pulsed laser (90 ps) at 2 mW/cm^2^ (20% power). Samples were imaged using a (CFI-SR-HP) Apochromat TIRF X100, NA of 1.49, oil-immersion objective (Nikon Instruments). Image stacks were generated by taking 1,000 serial images with an acquisition time of 4.8 ms per individual frames of 128 × 128 pixels (160 nm pixel size). The reflection light was detected using an avalanche photodiode (APD) with a band-pass filter of 650–720 nm.

TIRF images analysis: In each cell, a squared ROI of 121 pixels was chosen at the cell interface with the coverslip. Fluorescence intensity of each pixel in each image was normalized by dividing its intensity with the average intensity of that time-dependent image. The temporal fluctuations of the normalized fluorescence intensities were analyzed by DFT for each pixel in a ROI. The amplitudes of the DFT analyzes were then averaged for each frequency for all the pixels of an ROI to obtain the averaged DFT results of each ROI (or cell) in each condition.

### MSD Calculations

Jurkat cells were measured using a microscope in DIC mode, utilizing × 60 magnification and conditions that were described in detail in the previous sections. The measurements included repeated measurements every 0.3 s over a time window of 30 s. This measurement time allowed us to effectively avoid the constrains of the limited cell size (up to ∼10 μm) on diffusion. The cell image stacks were first converted to 8-bit images and thresholded (yielding binary images) to segment individual entities for tracking. Supplementary Figure 1 shows an example of DIC imaging of a representative Jurkat cell, before and after thresholding, on which particle tracking analysis was performed.

We defined thresholding levels according to the histogram of gray levels of the images. We noticed that a small range of thresholding values (in gray levels) were appropriate for segmentation, since too narrow threshold values caused fragmentation of the objects into isolated pixels, whereas threshold values that were too wide resulted in object contour thickening and unification. Analyzing the size distribution of the segmented objects, revealed that most of these objects were in the size range of intracellular vesicles or organelles (0.15 to ∼1.17 μm, average diameter 0.5 μm). Particles diameter were similar in cells before and after ATP depletion.

Further analyses of MSD statistics and fitting (“one term power series model fit”) were carried out using Matlab R2017b (MathWorks). Calculations of MSD values of intracellular objects were performed using the ImageJ plugin MultiTracker (The Kuhn lab; The University of Texas at Austin).

### Statistical Analyses

The acquired data was exported to Excel spreadsheets (Microsoft Office Professional plus 2010, Microsoft Inc., Redmond, Washington, United States) for graph and table presentation and for statistical analysis with Real Statistic Resource pack. Significance of differences between groups was calculated using Analysis of Variance (ANOVA) single factor function or *t*-test for paired two samples, with statistical significance set at *p* < 0.05.

The experimental results are shown from same-day experiments. The results were verified to be similar to those of 1–2 additional independent experiments (which are not shown).

## Data Availability Statement

The original contributions presented in the study are included in the article/Supplementary Material, further inquiries can be directed to the corresponding author/s.

## Author Contributions

ES supervised the research. ES and IW designed the research and wrote the manuscript. IW performed the research. Both authors contributed to the article and approved the submitted version.

## Conflict of Interest

The authors declare that the research was conducted in the absence of any commercial or financial relationships that could be construed as a potential conflict of interest.

## Abbreviations

DIC: Differential interference contrast
TIRF: Total internal reflection fluorescence
ATP: Adenosine triphosphate
MSD: Mean square displacement
IS: Immune synapse
PM: Plasma membrane
TCR: T cell receptor
MHC: Major histocompatibility complex
APC: Antigen presenting cell
FBM: Fractional Brownian motion
RWF: Random walk on fractal
CTRW: Continues time random walk
PSD: Power spectral density
BD: Brownian diffusion
DFT: Discrete Fourier transform
SSE: Sum of squared errors
PDF: Probability density function
SD: standard deviation
ROI: Region of interest
ASD: Amplitude spectral density
PFA: Paraformaldehyde
ANOVA: Analysis of Variance.
