# Supplementary material for: Spectral Analysis of ATP-Dependent Mechanical Vibrations in T Cells
Source: Front Cell Dev Biol. 2021 Jun 10;9:590655. doi: 10.3389/fcell.2021.590655 (PMC8222795; doi:10.3389/fcell.2021.590655)
Supplement: Supplementary file 1 [file Data_Sheet_1.docx]

**Supplemental Information**

**Spectral Analysis of ATP-dependent mechanical vibrations in T cells**

Ishay Wohl^1^, Eilon Sherman^1^*

*^1^Racah Institute of Physics, The Hebrew University, Jerusalem, 9190401, Israel*

* - corresponding author

*Possible correlations between DFT power fit parameters a and b and PDF (MSD) parameters* $K_{\alpha}$ *and* $\alpha$ *and ergodicity.*

Due to the possible relation between the DFT of a particle’s position change and the PDF of translocations, we analyzed the specific possible correlations between DFT powers fit parameters *a* and *b* in Eq. 1 and the diffusion parameters $K_{\alpha}$ and $\alpha$. Considering a diffusion process: an increase in $K_{\alpha}$ relates to an increase in the width of the PDF of translocations. This increase implies bigger translocations for most probabilities. Such a case implies elevated DFT amplitude results for most frequencies. This will produce higher values of the *a* parameter of the powers fit (in a general power function $y=a{/x}^{b}$, increasing *a* will increase all the function values). Therefore, it can be expected that $K_{\alpha}$ will be positively correlated to the *a* parameter of the power fit, as can be seen in the simulation presented in figure 1 e.

In ergodic sub-diffusion processes, $\alpha<1$ and the shape of the PDF of translocations may not be Gaussian. In such cases, the magnitude of translocations that correspond to the lower probabilities are relatively small in comparison to the magnitude of translocations for the same probabilities in a Gaussian PDF that characterizes Brownian diffusion. The DFT amplitudes that are related to this sub-diffusion PDF are characterized by relatively low amplitudes at low frequencies. The lower DFT amplitudes in the low frequencies range produces lower *b* values of the matching power fit. Note that in a general power function $y=a{/x}^{b}$, decreasing the value of the *b* parameter changes the shape of the function, where its elevated proximal (close to zero) part becomes less steeper and wider. For such ergodic sub-diffusion processes, the values of *b* are positively correlated to the $\alpha$ values. Following the same principles, the positive correlation of *b* and $\alpha$ values can be anticipated for the alternative condition of super-diffusion.

In non-ergodic anomalous diffusion processes, the $\alpha$ values may be relatively high and the shape of the PDF of translocation is non-Gaussian and may be non-symmetric. In such cases, the average magnitude of translocations corresponding to the lower probabilities (from both sides of the PDF graph) depends not only on the shape of the PDF of translocations but also on its symmetry. In this situation, the average magnitude of translocations corresponding to the lower probabilities of the PDF are no longer correlated primarily to the shape of the PDF of translocation and $\alpha$, but also depend on the non-ergodic process and the extent of asymmetry of the PDF. Accordingly, the correlation between $\alpha$ and *b* of the matched power fit may break due to a non-ergodic asymmetric process, esp. as a result of intracellular mechanical work.

*Imaging cell dynamics using DIC.*  See figure S1.

*DIC microscopy results of intracellular motion in live cells before and after blebbistatin treatment.* See figure S2.

**Supplemental Figures**


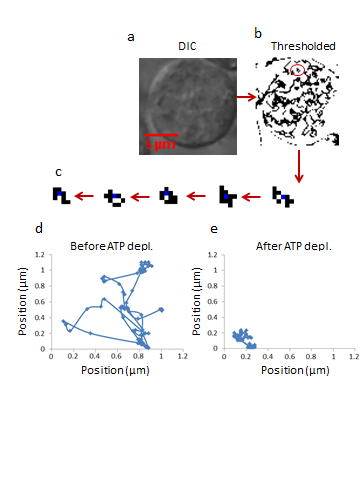


**Figure S1**. **Imaging cell dynamics using DIC**

(a) DIC image of a representative live Jurkat cell. (b) Image (a) after thresholding for object tracking. (c) An example of an individual tracked object. (d) Trajectories of an intracellular particle in an untreated cell (100 steps). (e) Trajectories of an intracellular particle in an ATP-depleted cell (100 steps).


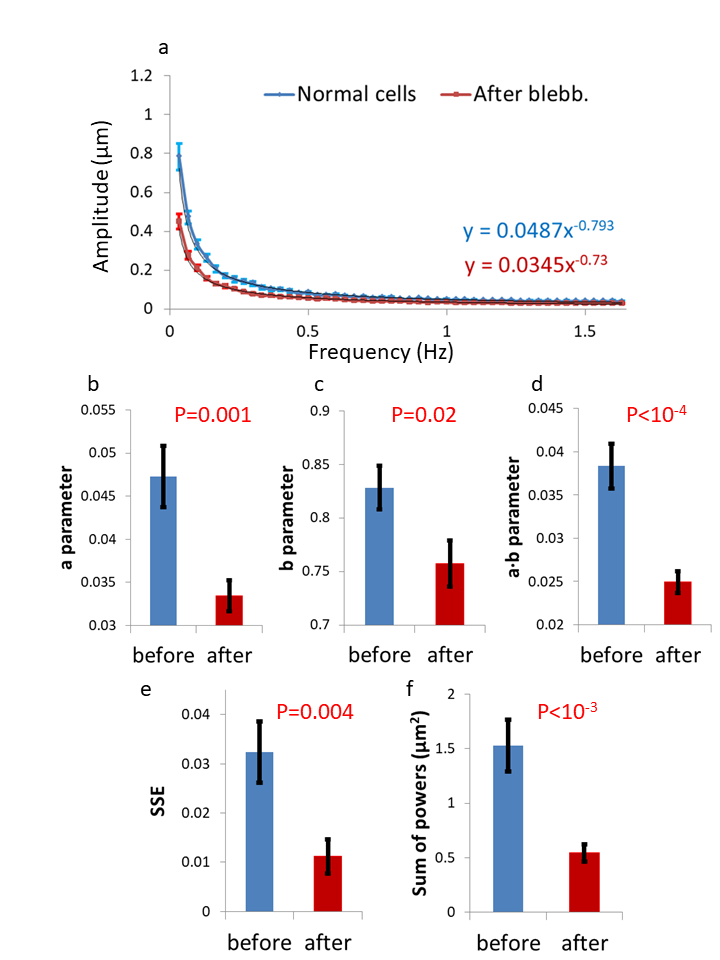


**Figure S2**. **DIC microscopy results of intracellular particles motion in live cells before and after 10μM blebbistatin treatment analyzed by ASD and PSD**

(a) ASD analysis results in (n=30) cells before (blue line) and after (red line) blebbistatin treatment and the corresponding power fit parameters. (b-e) Power fit parameters: *a*, *b*, *a∙b* and *SSE* for the ASD analysis results in the cells (in (a)) before and after blebbistatin treatment. (f) Power fit parameter ‘*Sum of powers*’ for the PSD analysis results in the cells (in (a)) before and after blebbistatin treatment. Error bars in panels b-f are SEM.
